# Kostenübernahme bariatrischer Operationen ohne Antragstellung – Erfahrungen in einem deutschen Adipositaszentrum

**DOI:** 10.1007/s00104-022-01690-z

**Published:** 2022-07-29

**Authors:** Isabel Auf der Maur, Daniel Gero, Gesa Kampmann, Tobias Prediger, Stefan Schopf, Jutta Peters, Jeannette Widmer, Jens Deerberg-Wittram, Christel Köhler-Hohmann, M. Bueter, Andreas Thalheimer

**Affiliations:** 1 Viszeral- und Transplantationschirurgie, Universitätsspital Zürich, Zürich, Schweiz; 2 Department Chirurgie, Spital Männedorf, Männedorf, Schweiz; 3 Allgemein‑, Viszeral‑, Endokrine und Unfallchirurgie, RoMed Klinik Bad Aibling, Bad Aibling, Deutschland; 4 RoMed Klinik Verbund Stadt und Landkreis Rosenheim, Rosenheim, Deutschland; 5 arztundklinikrecht.de/Dr. iur. Christel Köhler-Hohmann, Rechtsanwältin – Fachanwältin für Medizinrecht, Gilching bei München, Deutschland; 6 University Hospital Zurich: UniversitatsSpital Zurich, Zürich, Schweiz

**Keywords:** Medizinischer Dienst der Kassen (MDK), Krankenkassen, Sozialgericht, Adipositaschirurgie, Behandlungskosten, Medical service of health insurance, Health insurance, Social court, Bariatric surgery, Treatment costs

## Abstract

**Hintergrund:**

Nur ein kleiner Teil der Patienten mit morbider Adipositas hat in Deutschland Zugang zur derzeit effektivsten Therapie, der bariatrischen Operation. Ein wesentlicher Grund ist die restriktive Haltung der Krankenkassen hinsichtlich der Kostenübernahme.

**Ziel der Arbeit:**

Die Erfassung der postoperativen Rate an Kostenübernahmen durch die Krankenkassen ohne die derzeit übliche präoperative Antragstellung bei morbid adipösen Patienten, die eine leitliniengerecht indizierte bariatrische Operation in einem Adipositaszentrum in Bayern erhalten.

**Methodik:**

Der Prozess der postoperativen Kostenübernahmen wurde durch eine prospektive Datenbank im Zeitraum von 2 Jahren evaluiert. Die Fälle primärer Kostenübernahme wurden hinsichtlich Alter, BMI, Komorbiditäten und der Mitgliedschaft in einer bestimmten Krankenkasse korreliert. Abgelehnte Kostenübernahmen wurden bezüglich des weiteren anwaltschaftlichen und sozialgerichtlichen Prozesses verfolgt.

**Ergebnisse:**

Es wurden 188 Patienten ohne vorherige Antragstellung einer leitliniengerecht indizierten bariatrischen Operation zugeführt. Die primäre Kostenübernahme erfolgte in 76,6 % (*n* = 144). Es ergab sich keine Korrelation mit dem BMI, Komorbiditäten oder der Zugehörigkeit zu einer bestimmten Krankenkasse. Die Wahrscheinlichkeit einer postoperativen Kostenübernahme war bei Patienten über 40 Jahren signifikant wahrscheinlicher. Bei den Patienten ohne postoperative Kostenübernahme wurde in 7 Fällen eine außergerichtliche Einigung erreicht, 8 Fälle wurden rechtshängig, und 29 Fälle waren in anwaltschaftlicher Bearbeitung.

**Diskussion:**

Trotz der relativ hohen Rate primärer Kostenübernahmen zeigt sich auch in dieser Analyse die restriktive Haltung der Krankenkassen bezüglich der bariatrischen Operation mit entsprechendem ökonomischem Druck auf die Leistungserbringer. Die konsequente Umsetzung des antragsfreien Operierens erscheint notwendig, um den politischen Druck auf die Krankenversicherungen und Sozialgerichte zu erhöhen.

## Abstract (en)

**Background:**

Only a small proportion of patients with morbid obesity in Germany have access to the currently most effective treatment, bariatric surgery. A major reason for this is the restrictive attitude of health insurance companies regarding the reimbursement of costs.

**Objective:**

To record the postoperative rate of cost coverage by health insurance companies without the currently common preoperative application for morbidly obese patients who received a guideline-indicated bariatric surgery.

**Methods:**

The process of postoperative reimbursement was evaluated through a prospective database over a 2-year period. Cases of primary reimbursement were correlated with respect to age, BMI, comorbidities and membership of a specific health insurance company. Rejected coverage cases were followed up for further advocacy and social court process.

**Results:**

A total of 188 patients underwent bariatric surgery as indicated in the guidelines without prior application. Primary cost coverage was achieved in 76.6% (*n* = 144). There was no correlation with BMI, comorbidities or health insurance affiliation. Patients over 40 years of age were significantly more likely to be covered for costs. For patients without postoperative cost coverage, an out of court settlement was reached in 7 cases, 8 cases were heard by the social courts and 29 cases were still being processed by lawyers.

**Conclusion:**

Despite the relatively high rate of primary cost coverage, this analysis also shows the restrictive attitude of the health insurance companies regarding bariatric surgery with corresponding economic pressure on the service providers. The consistent implementation of application-free surgery seems necessary to increase the political pressure on health insurers and social courts.

## Hintergrund und Fragestellung

Aktuell stellt die bariatrische Chirurgie für Patienten mit einer morbiden Adipositas die effektivste Therapieoption dar. Neben der signifikanten Reduktion des Körpergewichtes und der damit einhergehenden Besserung verschiedener Adipositas-assoziierter Begleiterkrankungen steht die Vermeidung des Auftretens von kardiovaskulären, metabolischen und neoplastischen Erkrankungen mit dadurch bedingter Reduktion der Sterblichkeit im Vordergrund [1, 16]. Die Empfehlungen zur Chirurgie der Adipositas und metabolischer Erkrankungen sind in Deutschland in einer S3-Leitlinie zusammengefasst [7].

Unter Berücksichtigung der hohen Effektivität der Adipositaschirurgie erscheint es überraschend, dass in Deutschland weiterhin nur ein kleiner Teil der betroffenen Patienten mit morbider Adipositas Zugang zu dieser Therapie erhält [2]. Ein wesentlicher Grund für die im internationalen Vergleich geringe Anzahl bariatrischer Eingriffe in Deutschland ist die sehr restriktive Haltung der Krankenversicherungen hinsichtlich der Kostenübernahme für bariatrische Operationen [8]. Seit Beginn der laparoskopischen bariatrischen Chirurgie Ende der 90er- und Anfang der 00er-Jahre ist es daher üblich, vor Durchführung einer Operation bei den Kostenträgern einen schriftlichen Antrag auf Kostenübernahme einzureichen. Die häufige Ablehnung dieser Anträge – nach Beurteilung des Sachverhaltes durch den Medizinischen Dienst der Krankenkassen (MDK), in der Zwischenzeit in „Medizinischer Dienst“ umbenannt – erfolgt häufig auf wissenschaftlich nicht nachvollziehbaren Argumentationen, verhindert in vielen Fällen eine effektive und evidenzbasierte medizinische Therapie und stellt die Therapiefreiheit der behandelnden medizinischen Fachexperten in inakzeptabler Weise infrage. Hinzu kommt, dass die Beurteilung beantragter bariatrischer Operationen durch den MDK in unterschiedlichen Regionen Deutschlands häufig zu unterschiedlichen Ergebnissen führt, somit eine klare und reproduzierbare Bewertungsgrundlage nicht zu erkennen ist. Dies führt nicht nur zu einem sichtbaren Unterschied in der Anzahl der durchgeführten Operationen bezogen auf die Einwohnerzahl der einzelnen Bundesländer, sondern auch zum Eindruck der Willkür und Unvorhersehbarkeit [12]. Eine rechtliche Grundlage für die Antragstellung vor bariatrischer Operation besteht in Deutschland grundsätzlich nicht [18]. In diesem Spannungsfeld wurden bariatrische Eingriffe jahrelang teils sehr langwierig durch Patienten und Betroffene selbst mittels Widerspruchs- und Klageverfahren erstritten oder aber in Einzelfällen durch den Kostenträger genehmigt, ohne dass es einen begründbaren Zusammenhang mit der Schwere der Erkrankung oder der Dringlichkeit des Eingriffs gegeben hätte. Nachdem sich trotz intensiver politischer Arbeit und enger Kooperation der Adipositaschirurgen mit Patientenvertretern und -verbänden keine Besserung der Situation zeigte, hat die Chirurgische Arbeitsgemeinschaft Adipositaschirurgie (CAADIP) reagiert und im März 2018 im Rahmen der Initiative „OP ohne Kostenzusage“ allen adipositaschirurgischen Abteilungen empfohlen, medizinisch notwendige und leitliniengerecht indizierte Operationen ohne vorherige Antragstellung bzw. Kostenzusage durchzuführen [11]. Inwieweit dieser Empfehlung flächendeckend nachgekommen wurde, ist schwer einzuschätzen. Auch die Rate der postoperativen Kostenübernahmen durch die Krankenkassen ohne präoperative Antragstellung ist unklar.

Das Ziel dieser Untersuchung ist daher die Erfassung des Umfangs der postoperativen Kostenübernahmen in einem deutschen Adipositaszentrum in einem Zeitraum von 2 Jahren, in dem alle primären und sekundären bariatrischen Operationen ohne präoperative Antragstellung durchgeführt wurden.

## Methodik

Alle entsprechend der gültigen S3-Leitlinie indizierten primären bariatrischen Eingriffe an einem deutschen Adipositaszentrum (in Bayern, DGAV-Zertifizierung Kompetenzzentrum 2022) wurden im Zeitraum vom 01.01.2018 bis 31.12.2019 ohne präoperative Antragstellung zur Kostenübernahme durchgeführt. Bei allen Patienten unabhängig der Indikation zur Operation (bariatrische, metabolische oder primäre Indikation) war präoperativ eine leitliniengerechte konservative Therapie im Sinne einer mindestens 6‑monatigen Veränderung des Lebensstils unter Intensivierung der körperlichen Aktivität sowie einer im Zentrum durchgeführten Ernährungs- und psychotherapeutisch geführten Verhaltenstherapie erfolgt. In einer prospektiv geführten Datenbank wurden neben demografischen, patientenspezifischen und operationstechnischen Informationen auch Daten zur Kostenübernahme dokumentiert und im Rahmen der vorliegenden Kohortenanalyse ausgewertet. Bei allen Patienten besteht die Einwilligung zur wissenschaftlichen Bearbeitung der erhobenen Daten.

Nach erfolgter Operation erhielten die Kostenträger die Rechnung über die für die entsprechende DRG (Diagnosis Related Groups) kalkulierte Summe. Wurde diese vom Kostenträger beglichen, erfolgte keine weitere Kommunikation durch das Zentrum. Im Falle der Prüfung der Notwendigkeit der stationären Krankenhausbehandlung durch den Medizinischen Dienst der Krankenkasse (MDK) wurden die Behandlungsunterlagen vorgelegt. Wurde die Notwendigkeit des stationären Aufenthaltes für die bariatrische Chirurgie seitens des MDK nicht gesehen und dem Kostenträger somit keine Empfehlung zur Kostenerstattung gegeben, erfolgte seitens des Kostenträgers die Aufrechnung der kalkulierten Erlössumme mit einem anderen Fall desselben Kostenträgers. Ab diesem Moment wurde die Bearbeitung an eine Fachanwältin für Medizinrecht weitergeleitet. Auf der Basis der vorhandenen Patientendokumentation erfolgte dann grundsätzlich die weitere Kommunikation zwischen Fachanwältin und Kostenträger mit evtl. außergerichtlicher Einigung oder Einleitung eines sozialgerichtlichen Verfahrens.

Die Erlösanalyse erfolgte mit bis März 2021 gesammelten Daten. Die primäre Akzeptanz der Kostenrechnung durch den Kostenträger ohne Prüfung durch den MDK wie auch die primär positive MDK-Bewertung nach Auftrag durch den Kostenträger wurden als „primäre Kostenübernahme“ bewertet. Alle anderen Fälle (negative Bewertung durch den MDK, Aufrechnung seitens der Kostenträger, sozialgerichtliche Verfahren) wurden als „keine Kostenübernahme“ definiert. Die Wahrscheinlichkeit für „primäre Kostenübernahme“ in Korrelation mit dem Alter, dem BMI der Patienten, dem Vorhandensein von Adipositas-assoziierten Begleiterkrankungen und der Zugehörigkeit zu einem speziellen Kostenträger wurde mit einem binominalen Regressions-Modell berechnet. Die statistischen Berechnungen erfolgten mit R Software v. 4.1.2 (R Foundation for Statistical Computing, Wien, Österreich). Zum Vergleich der Häufigkeiten wurde der Pearson-Chi-Quadrat-Test genutzt, das Signifikanzniveau auf *p* < 0,05 festgelegt.

## Ergebnisse

### Patientendaten

Im untersuchten Zeitraum von 01.01.2018 bis 31.12.2019 wurde bei 188 allgemeinversicherten Patienten (78 % weiblich, 22 % männlich) mit einer Adipositas WHO Grad 3 eine bariatrische Operation ohne vorherige, präoperative Antragstellung zur Kostenübernahme durchgeführt. Das Patientenkollektiv zeigte ein Durchschnittsalter von 43,6 Jahren mit einem Durchschnitts-BMI von 48,6 kg/m^2^. Bei 163 Patienten (86,7 % des Gesamtkollektivs) waren eine oder mehrere Adipositas-assoziierte Begleiterkrankungen dokumentiert. Insgesamt wurde bei 101 Patienten (53,7 %) ein laparoskopischer proximaler Magenbypass (RYGB) und bei 87 Patienten (46,3 %) eine Sleeve-Gastrektomie (SG) durchgeführt. Mit 39,4 % war die Mehrheit der Patienten bei der AOK Bayern versichert, 14,3 % bei der Barmer Ersatzkasse, 11,7 % bei der Technischen Krankenkasse (TKK) und 34,6 % verteilten sich auf zahlreiche andere Kostenträger (Tab. 1).

**Table 1:** 

	(*n*)	(%)
Gesamtkollektiv (*n*)	188	
*Alter (Jahre)*	43,6 ± 11,4 (21–70)	
*BMI (kg/m*^*2*^*)*	48,6 ± 8,0 (35–84)	
*Geschlecht*		
Weiblich	147	78,2
Männlich	41	21,8
*Operationsart*		
RYGB	101	53,7
Sleeve	87	46,3
*Komorbidität*		
Diabetes	61	32,4
Art. Hypertonie	104	55,3
OSAS	54	28,7
Depression	31	16,5
GERD	16	8,5
PCO-Syndrom	8	5,8
Muskuloskeletal	70	37,2
*Krankenkasse*		
AOK	74	39,4
Barmer	27	14,4
Techniker	22	11,7
Andere	65	23,4

*RYGB* „Roux-en‑Y gastric bypass“, *OSAS* obstruktives Schlafapnoesyndrom, *GERD* „gastroesophageal reflux disease“, *PCO-Syndrom* „polycystic ovary syndrome“, *AOK* Allgemeine Ortskrankenkasse

Bei 144 Patienten aus dem Kollektiv (76,6 %) wurden postoperativ nach Einreichung der Kostenrechnung bei den Kostenträgern die Behandlungskosten für die bariatrische Operation direkt – ohne Prüfung durch den MDK oder nach vorheriger Prüfung durch den MDK und positiver Empfehlung zur Kostenübernahme – übernommen. Bei 44 Patienten (23,4 %) wurde die Kostenübernahme nach negativer Empfehlung des MDK abgelehnt und eine Aufrechnung der Behandlungskosten in die Wege geleitet. Bei 7 der 44 Patienten konnte durch anwaltschaftliche Vermittlung eine außergerichtliche Übernahme der Behandlungskosten erwirkt werden, bei 8 der 44 Patienten erfolgte die Einleitung eines Sozialgerichtsverfahrens. Davon waren 7 Verfahren noch laufend, 1 Fall zugunsten des Krankenhauses entschieden. Die restlichen 29 Patienten befanden sich in fachanwaltschaftlicher Bearbeitung. Somit wurde bei 18,2 % der Patienten mit einer primären Ablehnung der Kostenübernahme die sekundäre Kostenübernahme erstritten, die restlichen 81,8 %, dies entspricht 19,1 % des Gesamtkollektivs, waren noch nicht entschieden.

**Figure 1:**
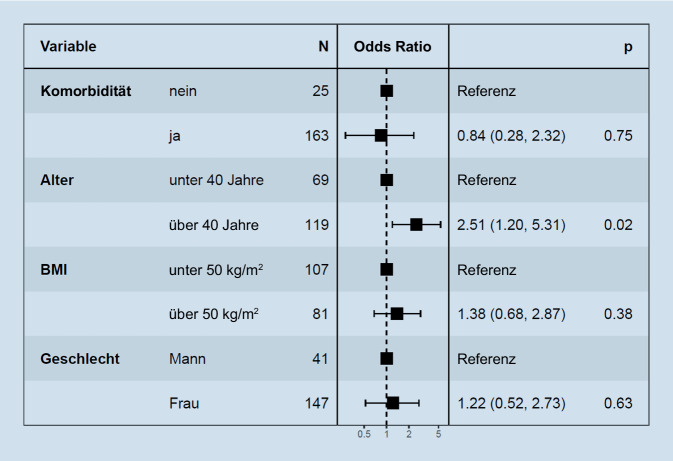

Die Wahrscheinlichkeit der primären Kostenübernahme war bei Patientenalter über 40 Jahre signifikant höher als bei Patienten unter 40 Jahren (*p* = 0,02). Das Vorhandensein von Adipositas-assoziierten Komorbiditäten, der BMI der Patienten oder das Geschlecht hatten keinen Einfluss auf die Wahrscheinlichkeit einer primären Kostenübernahme (Abb. 1).

**Figure 2:**
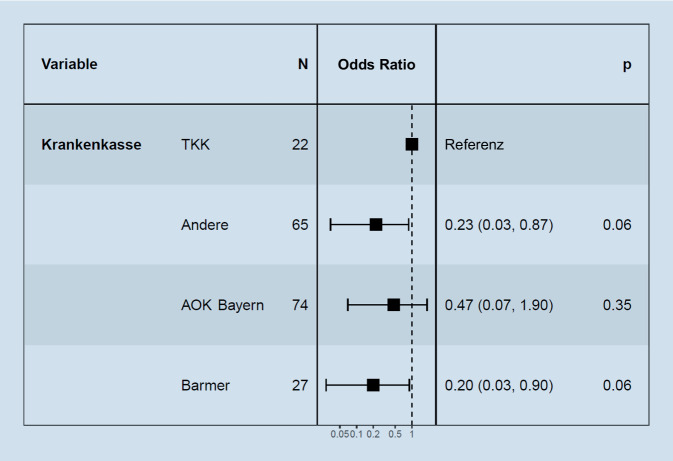

Die Zugehörigkeit zu einer bestimmten Krankenversicherung zeigte keinen rechnerischen Zusammenhang mit der Wahrscheinlichkeit der Kostenübernahme (Abb. 2).

## Diskussion

In dieser Kohortenanalyse wurden die Behandlungskosten von leitliniengerecht indizierten bariatrischen Eingriffen an einem deutschen Adipositaszentrum ohne präoperative Antragstellung zur Kostenübernahme bei knapp 77 % der Patienten von den Kostenträgern postoperativ übernommen. Die Wahrscheinlichkeit der Kostenübernahme war bei einem Lebensalter über 40 Jahre signifikant erhöht.

In zahlreichen prospektiven, randomisierten und kontrollierten Studien ist die Überlegenheit der bariatrischen Chirurgie gegenüber konservativer Therapie in der Behandlung der schweren Adipositas sowie deren Komorbiditäten gezeigt [1, 13, 15]. Gleichwohl erhält nur ein Bruchteil der Patienten, die sich durch ihr Körpergewicht oder durch metabolische Begleiterkrankungen grundsätzlich für eine operative Therapie qualifizieren, Zugang zur bariatrischen Chirurgie. Bezogen auf die Einwohnerzahl war das Missverhältnis zwischen effektiven Patientenzahlen und tatsächlich durchgeführten bariatrischen Eingriffen in Deutschland jahrelang besonders auffällig [2, 4]. Unzweifelhaft ist die weit verbreitete Stigmatisierung der Erkrankung „Adipositas“ sowohl bei Betroffenen als auch bei therapeutischen Spezialisten eine wesentliche Ursache für diese Situation [5, 9, 10]. Große Bedeutung für die fehlende flächendeckende Verbreitung der Adipositaschirurgie hat aber ebenso die restriktive Politik hinsichtlich der Übernahme der Behandlungskosten durch die Kostenträger. Vorschub für diese Haltung leistete ein Bundessozialgerichtsurteil aus dem Jahr 2003, in dem eine Erstattungspflicht der Krankenkassen dem Grundsatz nach zwar festgestellt wurde, die bariatrische Chirurgie zur Behandlung von Patienten mit einer schweren Adipositas allerdings nur als „Ultima Ratio“ bezeichnet wurde und diese nur dann in Betracht komme, wenn eine ganze Reihe von Bedingungen, wie etwa Erschöpfung konservativer Behandlungsmöglichkeiten und ausreichende Motivation seitens der Patienten, erfüllt seien [19]. Dies ließ Krankenkassen und auch Gerichten einen sehr weiten Interpretationsspielraum, der zu Rechtsunsicherheit und unklaren Entscheidungsprozessen führte. In der Folge etablierte sich das „Antragsverfahren“ zur Vorabgenehmigung einer bariatrischen Operation. Dies ist insofern interessant, da es sich bei einer bariatrischen Operation, die entsprechend den gültigen Leitlinien der Fachgesellschaften indiziert wird, um eine sog. „Regelleistung“ handelt, d. h. die Behandlung ist eine grundsätzlich von der Gesetzlichen Krankenversicherung (GKV) zu erbringende Leistung und bedarf keiner vorherigen Antragstellung. Der Anspruch des Versicherten hängt im Einzelfall von der medizinischen Indikation ab, welche von den behandelnden Ärzten festgestellt wird [18]. Schon in den Anfangsjahren der modernen bariatrischen Chirurgie zeichnete es sich ab, dass die Krankenkassen den ihnen durch das BSG-Urteil von 2003 überlassenen Interpretationsspielraum vollumfänglich ausnutzten. So berichten Gärtner et al. in einer deutschen Beobachtungsstudie, dass nur bei 31,6 % aller Patienten, bei denen fachärztlich und interdisziplinär die Indikation zur bariatrischen Operation gestellt und ein entsprechender Antrag zur Kostenübernahme eingereicht wurde, die Therapiekosten von den Krankenversicherungen übernommen wurden [8]. Auch die Publikation von wissenschaftlich basierten S3-Leitlinien unter Federführung der Chirurgen Arbeitsgemeinschaft Adipositaschirurgie (CAADIP) innerhalb der Deutschen Gesellschaft für Allgemein- und Viszeralchirurgie (DGAV) hat zu keiner wesentlichen und nachhaltigen Verbesserung dieser Situation geführt [7, 14]. Stetige politische Arbeit durch Patientenverbände und Leistungserbringer sowie eine auch juristisch zunehmende Akzeptanz der sog. primären Operationsindikation bei Patienten mit einem BMI > 50 kg/m^2^ [3] hat in der Folge dazu geführt, dass die Anzahl der Krankenhäuser, die adipöse Patienten ohne präoperative Antragstellung einer bariatrischen Operation zuführten, zugenommen hat. Das Kostenrisiko und das damit einhergehende Prozessrisiko verschiebt sich somit auf die Seite des operierenden Krankenhauses, da die Krankenkassen das Recht zur Überprüfung der stationären Behandlungsbedürftigkeit auf dem Boden der nach § 301 SGB V übermittelten Patientendaten sowie additiv durch Beauftragung einer nachgelagerten Abrechnungsprüfung durch den MDK haben [20]. Aktuell ist unklar, wie viele Krankenhäuser in Deutschland bariatrische Operationen ohne präoperative Antragstellung durchführen und welchem ökonomischen Risiko die leistungserbringenden Abteilungen durch die Verlagerung des Kostenrisikos ausgesetzt sind. Individuelle einzelne Erfahrungsberichte sind positiv und zeigen die grundsätzliche Machbarkeit dieses Vorgehens [20].

Die im Rahmen dieser Beobachtungsstudie analysierte Kohorte zeigt ein Durchschnittsalter von 43,6 Jahren und einen Durchschnitts-BMI von 48,6 kg/m^2^. Dies entspricht den Vergleichswerten im Rahmen deutscher Registerstudien zu anderen bariatrischen Fragestellungen und reflektiert die medizinische Versorgungsrealität in Deutschland genauso wie das Vorhandensein von Komorbiditäten in 86,7 % der Patientinnen und Patienten [17]. Die primäre Kostenübernahme für die bariatrische Operation in 76,6 % der Patienten erscheint hoch. Aktuelle Vergleichszahlen sind nicht publiziert, sodass die Einordnung und Interpretation der Zahlen schwierig sind. Noch einmal wird betont, dass diese Zahlen aufgrund der unterschiedlichen Bewertung bariatrischer Operationen durch den regional organisierten MDK nicht ohne Weiteres auf alle Regionen und Bundesländer in Deutschland übertragbar sind.

Die Wahrscheinlichkeit der Kostenübernahme in unserer Analyse ist unabhängig von BMI oder Komorbiditäten, zeigt aber eine signifikante positive Korrelation mit zunehmendem Patientenalter. Dies reflektiert wahrscheinlich weniger die erhoffte Überzeugung, dass bariatrische Chirurgie eine effektive Therapie für die Behandlung chronisch kranker Patienten ist, als vielmehr das leider immer noch weitverbreitete Denken, dass es sich bei der Operation um eine „Ultima Ratio“ nach Versagen aller konservativer Therapieinterventionen handelt. Neben der wirkungsvollen Therapie metabolischer Begleiterkrankungen zeigt die bariatrische Chirurgie überzeugende Effekte in der Vermeidung bekannter Adipositas-assoziierter Begleiterkrankungen sowie in der Reduktion maligner Erkrankungen im Verlauf und führt damit auch zur Verlängerung der Lebenserwartung betroffener Patienten [6]. Insofern ist es erstaunlich, dass die Kostenübernahme bei älteren Patienten offensichtlich eher erfolgt, da die genannten positiven Effekte bei früherer Durchführung der bariatrischen Operation auch früher zur Geltung kommen könnten.

Die Zugehörigkeit zu einer bestimmten Krankenversicherung zeigte keinen Zusammenhang mit der primären Genehmigung der Kosten, unabhängig davon welche der Krankenversicherungen als Referenzwert genommen wurde. Dies überrascht, da insbesondere die größte Krankenversicherung im Bundesland Bayern im Zusammenhang mit der Kostenübernahme bariatrischer Operationen negativ assoziiert ist [12]. Möglicherweise sind aber auch die Patientenzahlen in dieser Analyse zu gering, um entsprechende Zusammenhänge aufzuzeigen.

Die hohe Rate primärer Kostenübernahmen darf allerdings nicht darüber hinwegtäuschen, dass bei knapp einem Viertel aller Operationen die endgültige Kostenübernahme nicht gesichert ist. Sobald im Falle der Ablehnung der primären Kostenübernahme die Aufrechnung der kalkulierten Erlössumme mit einem anderen Fall desselben Kostenträgers erfolgte, wurden die Fälle an eine Fachanwältin für Medizinrecht weitergereicht. Unabhängig vom weiteren Verlauf (Übernahme der Kosten nach außergerichtlicher Einigung bzw. Vergleich, Übernahme der Kosten nach sozialgerichtlicher Auseinandersetzung) bedeutet dies eine signifikante krankenhausökonomische Belastung. Denn selbst wenn Einzelfallberichte darlegen, dass durch Intervention eines Fachanwaltes alle streitbaren Fälle zugunsten des Krankenhauses entschieden wurden [20], liegt das Kostenrisiko komplett aufseiten des leistungserbringenden Krankenhauses. Ein signifikanter Anteil dieser medizinischen Leistungen wird somit erbracht, ohne einen direkt verfügbaren Erlös zu generieren. Dies stellt bei steigenden Operationszahlen eine zunehmende ökonomische Belastung dar, die auch in der Diskussion mit dem Klinikleiter thematisiert werden muss. Letztendlich erscheint aber aktuell nur die konsequente und breite Anwendung des antragsfreien Operierens bariatrischer Patienten geeignet, den Druck auf die Krankenversicherungen und Sozialgerichte hochzuhalten, um damit eine für die betroffenen Patienten und die leistungserbringenden Krankenhäuser zufriedenstellende politische Lösung zu erzwingen.

## Fazit für die Praxis

- Bei leitliniengerechter Indikationsstellung für eine bariatrische Operation werden auch ohne präoperative Anträge für eine Kostenübernahme die Fallkosten in ca. 75 % der Fälle primär übernommen. Die primäre Kostenübernahme steht in keinem Zusammenhang mit dem Vorliegen von Komorbiditäten oder dem BMI und erscheint somit arbiträr. Die Wahrscheinlichkeit einer Kostenübernahme ist bei größerem Patientenalter erhöht und spiegelt somit die noch immer in vielen Köpfen existente „Ultima Ratio“-Denkweise wider.
- Durch die zeitliche Verzögerung der Kostenübernahme von bariatrischen Operationen, die oftmals erst nach anwaltschaftlicher Intervention bezahlt werden, kann eine signifikante ökonomische Belastung einer leistungserbringenden Abteilung entstehen. Hier ist der Schulterschluss zwischen Klinikleitung und bariatrischen Chirurgen notwendig, um die entsprechenden und dringend benötigten politischen Veränderungen in Deutschland zu bewirken.
